# Strategies for Aortic Root Measurement in Patients Undergoing Surveillance for Thoracic Aortic Disease

**DOI:** 10.3390/jcm15093349

**Published:** 2026-04-28

**Authors:** Asama Rana, Irbaz Hameed, Sedem Dankwa, Danial Ahmad, Cameron Best, Sem Asmelash, Jose Anzueto, Sriharsha Talapaneni, Michela Cupo, Akbar Bazarbaev, Shiv Verma, Chanseo Lee, Titilayo Oden Shobayo, Prashanth Vallabhajosyula

**Affiliations:** 1 Division of Cardiac Surgery, Department of Surgery, Yale School of Medicine, New Haven, CT 06510, USA; asama.rana@yale.edu (A.R.); irbaz.hameed@yale.edu (I.H.); sedem.dankwa@yale.edu (S.D.); bazarbaevakbar99@gmail.com (A.B.);; 2 Yale Aortic Institute, Yale Pulmonary Thromboendarterectomy Program, Division of Cardiac Surgery, Yale School of Medicine, 330 Cedar Street BB204, New Haven, CT 06520, USA

**Keywords:** aortic root measurement, Laplace diameter, thoracic aortic aneurysm and dissection, surgical threshold, risk stratification, computed tomography imaging, aortic root geometry

## Abstract

**Objectives**: Several measurement techniques have been proposed to address the non-circular geometry of the aortic root. The Laplace diameter metric incorporates the cloverleaf anatomy of the aortic root and is derived via measurement of sinus-to-commissure lengths with subsequent doubling of the largest radius from the center. This study compares the conventional sinus-to-sinus with the novel Laplace method for sizing the aortic root and quantifying its implication on surgical decision-making. **Methods**: Patients undergoing surveillance at a high-volume aortic center were categorized by aortic root morphology as nondilated, non-syndromic dilated, bicuspid aortic valve and Marfan syndrome. Aortic root diameters by sinus-to-sinus and Laplace diameter methods were measured on computed tomography, compared using paired *t*-tests, and correlated using Spearman rank coefficients. **Results**: Of the 1297 patients assessed, 530 were included in the final analysis (nondilated *n* = 113, non-syndromic dilated *n* = 347, bicuspid aortic valve *n* = 50, Marfan syndrome *n* = 17). Aortic root diameters were significantly larger by Laplace than sinus-to-sinus diameter across all groups (sinus-to-sinus: 1.9 ± 5.5 mm; Laplace: 44.9 ± 7.0 mm; 95% confidence interval 2.72–3.34; *p* < 0.0001). Although Laplace and sinus-to-sinus diameter were correlated (Spearman *r* = 0.6789, 95% CI 0.6–0.7; *p* < 0.0001), the relationship was non-linear (R^2^ = 0.492). Laplace diameter increased the proportion of patients meeting surgical thresholds (2022 AHA/ACC guidelines) versus sinus-to-sinus: nondilated 0% vs. 1.77%, non-syndromic dilated 4.9% vs. 25.1%, bicuspid aortic valve 10.0% vs. 26.0%, and Marfan syndrome 23.5% vs. 52.9%. **Conclusions**: On average, Laplace diameter exceeded sinus-to-sinus diameter by 3 mm and would extend surgical eligibility to an additional 21% of patients under current guidelines.

## 1. Introduction

Thoracic aortic aneurysms and dissections (TAADs) are silent, yet fatal conditions associated with significant morbidity and mortality. Current guidelines suggest that aortic roots greater than 40 mm for men and 34 mm for women indicate aneurysmal disease which warrant surveillance [1]. Aortic root size greater than 50 mm is a trigger for surgical repair according to the current 2022 American College of Cardiology/American Heart Association (ACC/AHA) guidelines [2].

Based on current knowledge, the propensity of an aortic aneurysm to rupture or dissect hinges on the balance between the tensile strength of the aortic wall and the mechanical stress within the lumen. When the latter surpasses the wall’s capacity, rupture and dissections occur [3,4]. Aortic diameter has been shown to correlate closely with wall stress. The aortic root with its cloverleaf anatomy for the three-cusp aortic valve (TAV) lacks a universally accepted definition for diameter measurement. Current guidelines endorse multiple measurement techniques for the aortic root, each yielding significantly different results [2]. For instance, measuring from the deepest point of one sinus to the opposing commissure often underestimates true aortic root diameter, whereas measuring across sinuses, sinus to sinus diameter (SSD) captures a more comprehensive dimension [5]. This variability introduces inconsistencies in clinical management and research, as aortic centers differ in their choice of measurement technique. In 2022, ACC/AHA guidelines recommended using the SSD technique for root diameter measurement [2]. To address this challenge, Ban et al. introduced the Laplace diameter (LD), a novel measurement technique designed to account for the complex cloverleaf anatomy of the TAV aortic root [6]. Based on finite element computational simulations, the LD provides a more accurate estimation of wall stress compared to conventional methods, thereby potentially offering a more geometrically consistent basis for risk stratification, though its clinical superiority over conventional methods remains to be validated in prospective outcome studies. Their preliminary findings suggest that the Laplace diameter consistently produced larger measurements than traditional techniques. While compelling, the results are limited in statistical power.

In this study, we retrospectively apply the LD method using computed tomography (CT) imaging data from 1300 patients under surveillance for thoracic aortic disease. By comparing the Laplace diameter to conventional SSD measurements, we aimed to evaluate the differences between these measurement techniques and assess the clinical implications of adopting the Laplace method versus the SSD method in risk assessment and surgical decision-making.

## 2. Methods

### 2.1. Patient Selection

Surveillance for thoracic aortic disease at a high-volume aortic center was retrospectively evaluated. Patients undergoing CT imaging prior to operative intervention of aortic root disease were excluded from the study. Patients without cardiac radiologist or surgeon measurements of SSD were excluded from the study.

### 2.2. Study Design

Patients were grouped as nondilated root (NDR) [having an SSD < 40 mm for men and <34 mm for women], non-syndromic dilated root (NSDR) [SSD > 40 mm for men and >34 mm for women], bicuspid aortic valve (BAV) and Marfan’s syndrome (MS).

**Figure 1:**
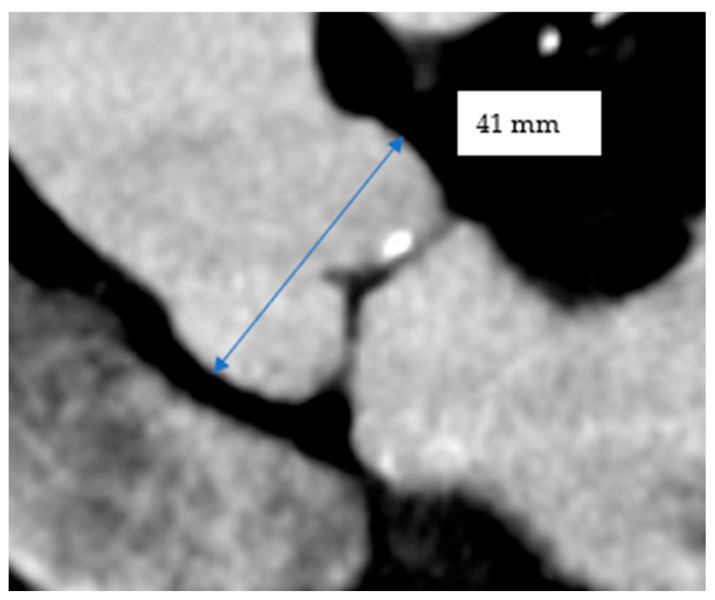
Measuring the aortic root in the coronal view.

For each patient, aortic root diameters were measured using CT imaging. The SSD was measured in the coronal view of the aorta where the two deepest sinuses are visible and measured from end to end (Figure 1) [7].

The aortic root size is estimated with a double arrow traversing each sinus-to-sinus length to give our sinus-to-sinus diameter.

**Figure 2:**
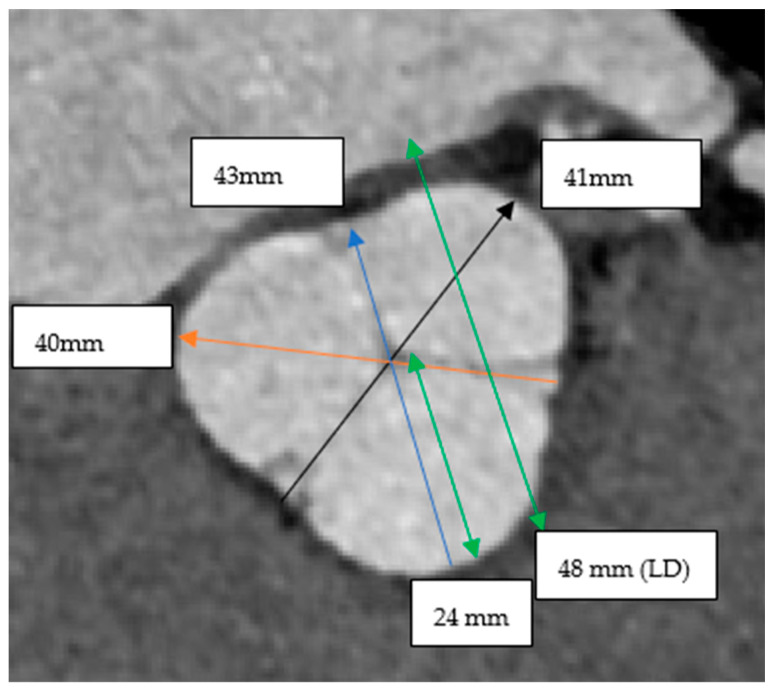
Measuring Laplace diameter with the aortic root in a coronal oblique view.

The LD was derived from an oblique coronal view of the aortic root, where 3 lines are made from the deepest sinus of Valsalva to the opposite commissure. This creates an intersection of three lines from which the largest radius is measured and doubled to find the LD (Figure 2) In theory, the LD incorporates all the sinus of Valsalva to yield an accurate size of the aortic root.

The same aortic root is used to calculate the Laplace diameter. Each sinus-to-commissure length is measured, as seen with each arrow: black (41 mm), blue (43 mm) and orange arrow (40 mm). The largest radius from the center is then measured, as seen with the green double arrow (24 mm), which is then doubled to obtain the Laplace diameter (48 mm).

### 2.3. Statistical Analysis

Continuous variables were summarized as medians with interquartile ranges and compared using analysis of variance (ANOVA) with Tukey’s multiple comparisons post hoc test as needed; normality was assessed using the Shapiro–Wilk test and, given the small Marfan syndrome subgroup (*n* = 17), non-parametric approaches were applied where normality could not be confirmed. Categorical variables were summarized as counts and percentages and compared using Chi-square test or Fisher’s exact test, as appropriate. SSD and LD measurements were compared using paired *t*-tests or Wilcoxon matched-pairs signed rank tests, as determined by Shapiro–Wilk normality testing, and their relationship assessed using the Spearman rank correlation coefficient. Agreement was established between SSD and LD. A two-tailed *p*-value < 0.05 was considered statistically significant. All analyses were performed using GraphPad Prism 10.4.1 (GraphPad Software, Boston, MA, USA).

## 3. Results

### 3.1. Patients’ Characteristics

A total of 530 patients meeting the inclusion criteria were evaluated. The study population comprised majority males (76.5%) with a mean age of 65.3 years (SD 11.6) and a mean BMI of 29.9 kg/m^2^ (SD 5.75). The most prevalent comorbidities were hypertension (49.7%), dyslipidemia (37.8%) and history of cerebrovascular disease (35.1%) with no statistically significant differences among the groups (*p* > 0.05). Of note, baseline atrial fibrillation (17.5%) was statistically significantly different among the groups with the highest prevalence seen in the Marfan syndrome group and the lowest prevalence in the nondilated root group (29.4% vs. 7.1%, *p* < 0.05) (Table 1).

### 3.2. Overall Comparison Between LD and SSD

**Figure 3:**
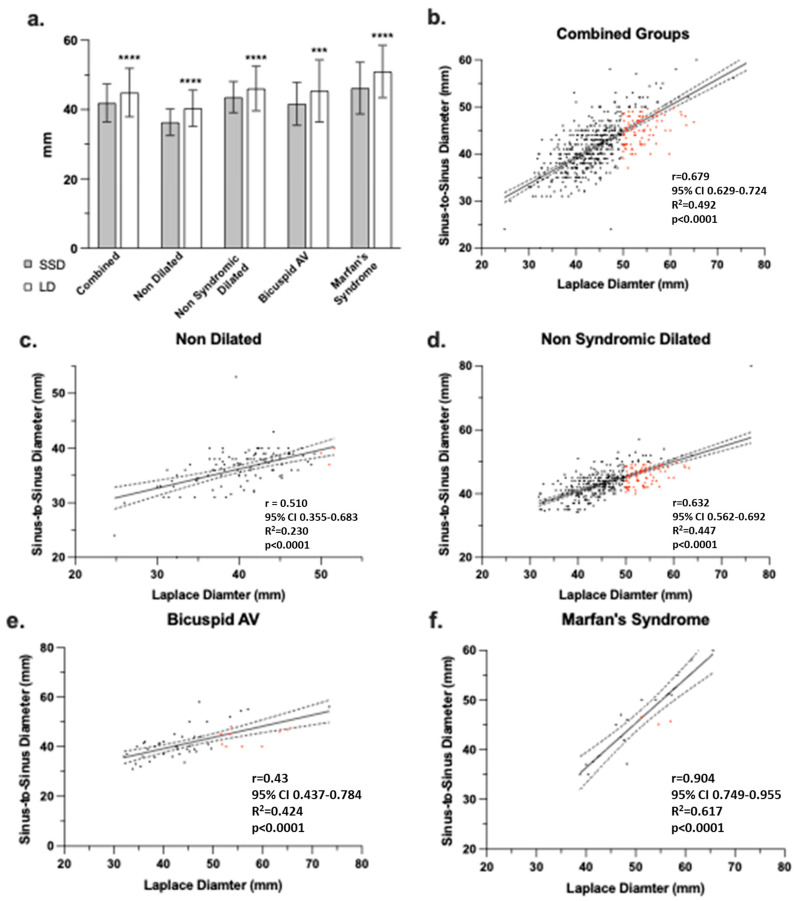
Laplace is larger than and correlates with sinus-to-sinus diameter measurements. (**a**) Aortic roots measured as either sinus-to-sinus (SSD, grey) or Laplace (LD, white) diameters for all patients (combined) and then by group. Intra-group comparisons performed via Wilcoxon matched-pairs signed rank or paired *t* tests as determined by normality testing; *p* values < 0.0001 indicated by **** and *p*-values < 0.0001 indicated by ***. Sinus-to-sinus vs. Laplace diameter measurements for (**b**) all patients (combined), (**c**) nondilated, (**d**) non-syndromic dilated, (**e**) bicuspid AV, and (**f**) Marfan’s syndrome groups. Spearman *r* with 95% CI, R^2^, and *p* values reported for each correlation. A solid line represents the simple linear regression of each plot and dashed lines represent the 95% CI. Red data points indicate those patients that would qualify for intervention using Laplace but not sinus-to-sinus diameter.

Overall, patient aortic root measurements were significantly greater when measured by LD compared to SSD for all patients (Figure 3a. *n* = 530, SSD: 41.9 ± 5.5 mm, LD: 44.9 ± 7.0 mm, mean difference (MD) of 3 mm, 95% confidence interval (CI) 2.72–3.34, *p* < 0.0001). While the LD and SSD were correlated (Figure 3b. Spearman *r* = 0.6789, 95% CI 0.6 to 0.7, *p* < 0.0001), this correlation was not linear (R^2^ = 0.492). 

### 3.3. Comparison Between LD and SSD Across the Groups

In the NDR group, patients’ aortic roots were larger when assessed by LD compared to SSD (Figure 3a. *n* = 113, SSD: 36.3 ± 3.8 mm, LD: 40.4 ± 5.2 mm, MD of 4.1 mm, 95% CI 3.21–5.03, *p* < 0.0001). Although the NDR group’s LD and SSD were correlated (Figure 3c. Spearman *r* = 0.510, 95% CI 0.4 to 0.6, *p* < 0.0001), this correlation was not linear (R^2^ = 0.250).

In the NSDR group, aortic root measurements were also greater when assessed by LD compared to SSD (Figure 3a. *n* = 348, SSD: 43.6 ± 4.5 mm, LD: 46.1 ± 6.5 mm, MD of 2.5 mm, 95% CI 2.3–3.2, *p* < 0.0001). In this group, the LD and SSD were correlated (Figure 3d. Spearman *r* = 0.632, 95% CI 0.6 to 0.7, *p* < 0.0001); however, this correlation was not linear (R^2^ = 0.447).

In the BAV group, aortic root measurements were similarly greater when assessed by LD compared to SSD (Figure 3a. *n* = 50, SSD: 41.7 ± 6.2 mm, LD: 45.4 ± 9.0 mm, MD of 3.7 mm, 95% CI 1.84–5.58, *p* = 0.0004). These measurements were correlated (Figure 3e. Spearman *r* = 0.43, 95% CI 0.4 to 0.8, *p* < 0.0001) but not linear (R^2^ = 0.424).

Finally, in the MS group like the other groups, aortic root measurements were greater when assessed by LD compared to SSD (Figure 3a. *n* = 17, SSD: 46.2 ± 7.5 mm, LD: 51.0 ± 7.6 mm, MD of 4.8 mm, 95% CI 3.15–6.48, *p* < 0.0001). In this group, the two measurements correlated (Figure 3f. Spearman *r* = 0.90, 95 CI 0.8 to 0.9, *p* < 0.0001) and approached linearity (R^2^ = 0.817).

### 3.4. Agreement Between LD and SSD

Since LD and SSD were significantly correlated in all groups but yielded systematically different aortic root measurements, Bland–Altman analysis was performed to assess agreement and quantify systematic differences between modalities.

**Figure 4:**
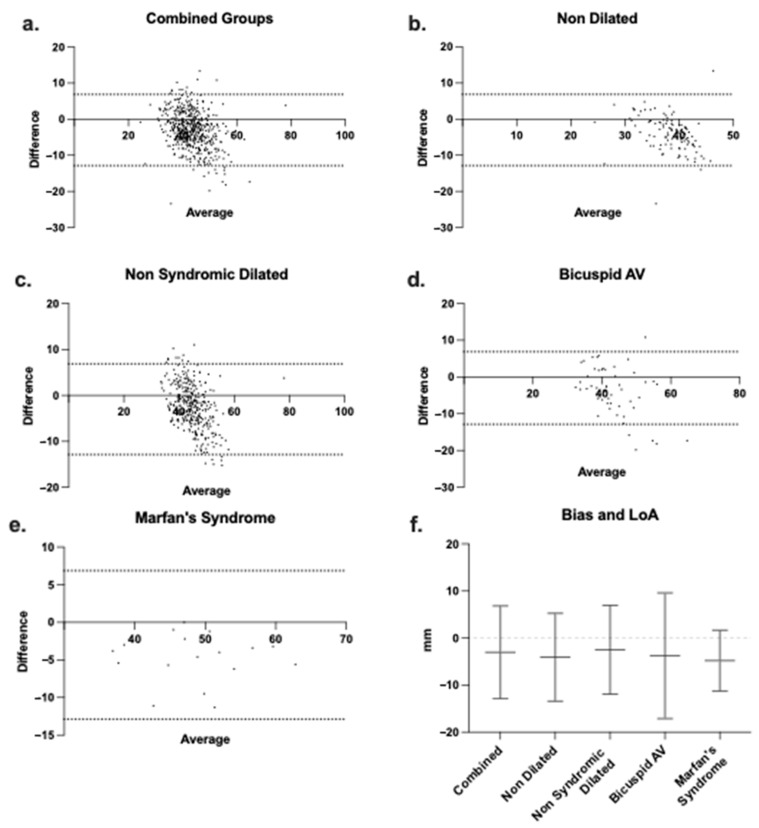
Laplace agrees with sinus-to-sinus measurement and yields consistently larger (biased) aortic root diameter. Bland–Altman agreement analysis (difference between Laplace and sinus-to-sinus diameter measurements vs. mean) for (**a**) all patients (combined), (**b**) nondilated, (**c**) non-syndromic dilated, (**d**) bicuspid AV, and (**e**) Marfan’s syndrome groups. Dashed horizonal lines represent the limits of agreement (LoA), or the values within which 95% of the differences between the Laplace and sinus-to-sinus measurements would be expected. (**f**) The mean bias (discrepancy) between Laplace and sinus-to-sinus diameter measurement and the limits of agreement (LoA) between the two modalities for each patient group.

Overall, LD measurements were larger by 3.0 ± 5.0 mm (mean difference, Figure 4a,f. 95% limits of agreements (LoA) −12.9 to 6.9 mm) relative to SSD in all patients, independent of aortic phenotype (Combined) (Table 2), and this trend was consistent across groups.

In the NDR group, LD measurements were 4.1 ± 4.8 mm greater than SSD (Figure 4b,f. 95% LoA −13.4 to 5.3 mm). In the NSDR group, LD measurements were 2.5 ± 4.8 mm greater than SSD measurements (Figure 4c,f. 95% LoA −11.9 to 6.9 mm). Similarly, LD measurements were 3.7 ± 6.8 mm larger than SSD measurements in the BAV group (Figure 4d,f. 95% LoA −17.11 to 9.6 mm) and 4.8 ± 3.3 mm larger in the MS group (Figure 4e,f. 95% LoA −11.3 to 1.7 mm).

In all groups, interpretation was limited by the large LoA of the modality reported. This is likely attributed to the limits of resolution of current cross-sectional imaging technologies and our inclusion of both contrasted and non-contrasted scans. In summary, on average, regardless of root phenotype, LD measurement yields a root diameter 2.5 to 4.8 mm larger than the SSD.

### 3.5. Surgical Intervention Candidacy

In all groups, aortic root measurements by the LD, compared to SSD, led to more patients exceeding the diameter thresholds to qualify for surgery—NDR: 0% vs. 1.77%, NSDR: 4.9% vs. 25.1%, BAV: 10.0% vs. 26.0%, and MS: 23.5% vs. 52.9% (Table 2).

## 4. Discussion

The underlying goal of various imaging techniques used to measure aortic dimensions [8,9] is accurate anatomic characterization with subsequent assessment of thresholds for prophylactic intervention, aiming to mitigate the risk of catastrophic aortic complications such as dissection and rupture in at-risk aortas, particularly those with aneurysms [10]. The mortality and morbidity burden for these events is very high and likely underreported, especially in cases of sudden death [11,12]. The aortic root warrants special attention because of its non-circular geometry, in contrast to the more cylindrical ascending and descending thoracic aorta [13]. The cylindrical configuration of the latter partially conforms to the law of Laplace, which relates wall stress to pressure and vessel radius which is an essential determinant of aortic pathology once wall stress exceeds aortic tissue’s structural integrity [14]. In their introductory paper on the “Laplace Diameter,” Ban et al. conceptualized it as an extension of the law of Laplace to the aortic root, accounting for its complex, non-circular morphology [6].

In this study, we compared the LD and the conventional SSD for sizing the aortic root in patients undergoing surveillance for thoracic aortic disease. The large sample size, coupled with the use of CT scans to ensure precise measurements [7], enhances the validity of our findings. Additionally, inclusion of various patient phenotypes allows for a comprehensive understanding of measurement differences which align with imaging recommendations [15]. We found the LD to be significantly larger than SSD across all patient groups: NDR with a mean difference of 4.1 mm, NSDR with 2.5 mm, BAV with 3.7 mm, and MS with 4.8 mm (Table 2). The results in our study demonstrate that the LD consistently measures the aortic root larger than the conventional SSD across all patient groups, with statistically significant mean differences (MD) ranging from 2.5 mm to 4.8 mm (Table 2, *p* < 0.0001 for all). These differences are visually evident in Figure 3a, which highlights LD’s larger measurements across all cohorts. Correlation analysis reveals a significant relationship between LD and SSD, though it varies by group, suggesting LD may better capture the complex aortic root geometry in syndromic conditions. Bland–Altman analysis confirms LD’s consistent overestimation, though wide limits of agreement indicate variability (Figure 4f). Clinically, these discrepancies may affect surgical decision-making per the 2022 ACC/AHA guidelines (>50 mm threshold) [2]. The clinical significance of this variability is most apparent when considered in the context of the 50 mm surgical threshold: given LoA ranging from approximately −11 to +10 mm across groups, a patient measured near this threshold by one method could plausibly fall on either side depending on measurement approach, scan protocol, or observer. This is reflected directly in the reclassification data: in the NSDR group, for example, LD reclassified 25.1% of patients as above threshold compared to 4.9% by SSD—a difference that cannot be attributed to systematic bias alone and that underscores the need for prospective outcome data to determine which measurement more accurately identifies patients truly at elevated risk.

Our results are consistent with those from Ban et al., who first introduced LD as a metric derived from the extended law of Laplace to account for the complex cloverleaf anatomy of the aortic root [6]. Their study involving 106 patients also found LD to be larger than conventional measurements such as the mid-sinus to mid-sinus, depth to commissure, and coronal dimensions, suggesting on theoretical grounds that LD may more closely reflect wall stress. Our study extends these measurement observations to a larger cohort of 530 patients across diverse phenotypic groups; whether this translates to improved clinical risk stratification requires prospective validation against aortic outcomes. The classification of normal versus dilated roots in our study was informed by reference values from Pham et al., who determined normal roots to be <40 mm for men and <34 mm for women [1]. Sizes larger than these aforementioned values would correlate with a dilated root, hence the delineating factor for the NDR and NSDR groups.

From a clinical perspective, the larger measurements obtained with LD may translate to earlier or more frequent surgical interventions; whether this would confer clinical benefit or risk remains unknown in the absence of outcome data, and the possibility of overtreatment cannot be excluded. Given the mean differences observed (ranging from 2.5 mm to 4.8 mm), even small changes could push patients over the surgical threshold of 50 mm as defined by the 2022 ACC/AHA guidelines [2]. For patients with bicuspid aortic valves, the 2020 ACC/AHA guideline recommends intervention at root sizes of ≥50 mm or ≥45 mm with additional risk factors (i.e., hypertension, diabetes, hyperlipidemia, etc.), highlighting the relevance of accurate measurement in this population [16]. This is supported by Michelena et al., who noted the increased risk of aortic complications, such as aortic dissections, in BAV patients, further emphasizing the importance of accurate measurements [17]. Discrepancies in measurement methods can complicate clinical decision-making leading to increased morbidity and mortality from the aforementioned complications, as discussed by Elefteriades et al., emphasizing the need for standardized approaches [5].

To address these implications, future research should focus on prospective studies to evaluate the predictive value of LD for aortic complications, comparing it with SSD and other measurement techniques such as area-based or three-dimensional assessments. Complementing geometric measurements with CT-based tissue characterization—such as quantification of aortic valve fibrosis and calcification as demonstrated by Grodecki et al. [18]—represents a promising avenue for integrating biological wall properties with structural measurement strategies, potentially improving the prognostic precision of LD-based risk stratification. Standardization of LD measurement in clinical practice is also crucial, potentially requiring consensus guidelines from professional societies to ensure consistency and accuracy, as suggested by the recommendations for cardiac chamber quantification [19]. Additionally, validating LD in different populations, including those with other genetic aortic diseases, will enhance its applicability and generalizability, particularly given the normal values provided by Pham et al. for diverse demographics [1]. These findings demonstrate that LD reclassifies a substantially larger proportion of patients as meeting geometric surgical thresholds across all groups, most markedly in MS and BAV. Whether this reclassification identifies patients at genuinely elevated risk of adverse events—or reflects measurement-driven threshold crossing without corresponding biological risk—cannot be determined from the present data and requires prospective outcome validation. It is important to emphasize, however, that the increased proportion of patients meeting surgical thresholds under LD does not imply that these patients carry correspondingly higher risk of aortic dissection or rupture. Threshold crossing driven by a larger geometric measurement is not equivalent to evidence of elevated biological risk or undertreated disease. In the absence of longitudinal outcome data linking LD measurements to adverse aortic events, the reclassification observed here should be interpreted as a measurement-driven phenomenon that defines the scope and hypothesis-generating value of the present study, rather than as a direct indication for intervention.

## 5. Limitations

Since this is a retrospective analysis, there is the risk of selection bias despite our efforts to control for known confounders. We lack accurate data on clinical outcomes of patients such as aortic dissection or rupture to validate whether LD is a better predictor of adverse events than SSD. This is crucial because while LD may provide larger measurements, it is essential to determine if these measurements correlate more accurately with the risk of clinical events, as emphasized by Elefteriades et al. [20]. Lastly, our study did not assess inter-observer variability in SSD or LD measurements, which could affect reproducibility, although we followed the standardized image measurement protocol as referenced by Ban et al. [6]. Previous studies have highlighted variability in measurements between different imaging modalities and observers, emphasizing the need for standardized measurement techniques, as shown by Plonek et al. [21]. Additionally, not all patients in this cohort underwent ECG-gated or contrast-enhanced CT acquisitions; imaging was obtained per institutional clinical protocol during routine aortic surveillance, and non-gated and non-contrasted scans were included where clinically indicated. ECG-gating is recognized as the optimal technique for aortic root measurement, minimizing motion artifact across the cardiac cycle, and its absence in a subset of patients may have introduced additional variability into root diameter measurements. The wide limits of agreement observed in the Bland–Altman analysis are at least in part attributable to this scan protocol heterogeneity. We acknowledge this as a limitation; however, deliberately excluding non-gated scans would substantially reduce sample size and introduce selection bias toward patients with more advanced or symptomatic diseases. Importantly, in real-world aortic surveillance practice, non-gated CT acquisitions are common, and characterizing the behavior of both SSD and LD measurements in this pragmatic setting has direct clinical relevance. Future prospective studies should standardize ECG-gated, contrast-enhanced CT acquisition to allow more precise comparison of these measurement strategies. Finally, the Marfan syndrome subgroup (*n* = 17) is small, limiting the statistical power of subgroup-specific inferences; while non-parametric analyses were applied in this group as determined by normality testing, findings in this subgroup should be interpreted with caution and require validation in larger syndromic cohorts. Furthermore, while the theoretical foundation of LD rests on finite element computational simulations that model wall stress based on geometric assumptions, this framework does not incorporate tissue-level material properties such as medial fibrosis, calcification, or extracellular matrix degeneration. These histopathological features can meaningfully alter aortic wall mechanics and failure thresholds, yet tissue-level validation—whether through surgical specimens or ex vivo biomechanical testing—was not available in the present cohort. Emerging CT-based approaches for quantifying aortic wall fibrosis and calcification, as described by Grodecki et al. [18], may offer a path toward integrating structural tissue characteristics with geometric measurement strategies in future studies, potentially refining the risk stratification value of LD beyond what geometry alone can provide.

## 6. Conclusions

Our study demonstrates that the Laplace diameter is significantly larger than the conventional sinus-to-sinus diameter in measuring the aortic root with potential implications for surgical decision-making in patients with thoracic aortic disease. Further research is needed to validate the clinical relevance of LD and establish its role in managing these patients.

## Figures and Tables

**Table 1:** Demographics/patient selection table. Body mass index (BMI), diabetes mellitus (DM), chronic kidney disease (CKD), chronic pulmonary obstructive disease (COPD), cerebrovascular disease (CVD), peripheral vascular disease (PVD), coronary artery disease (CAD), nondilated root (NDR), non-syndromically dilated root (NSDR), bicuspid aortic valve (BAV), Marfan’s syndrome (MS).

Variable	NDR % (114)	NSDR % (348)	BAV % (51)	MS % (17)	Total %	*p*-Value
Age, mean (SD)	66.4 (11.4)	66.5 (10.70)	58.2 (12.7)	54.5 (14.4)	65.3 (11.6)	<0.0001
BMI, mean (SD)	27.5 (5.38)	30.8 (5.61)	30.6(6.21)	25.3 (3.92)	29.9 (5.75)	0.03
Male	79 (69.9)	273 (78.7)	40 (80.0)	11 (64.7)	403 (76.5)	0.15
DM (%)	21(18.6)	68 (19.6)	7 (14.0)	1 (5.88)	97 (18.4)	0.43
Hypertension	60 (53.1)	177 (51.0)	22 (44.0)	3 (17.6)	262 (49.7)	0.09
CKD	10 (8.85)	13 (3.75)	1 (2.00)	0 (0.00)	24 (4.55)	0.19
Smoking history	1 (0.88)	7 (2.02)	0 (0.00)	0 (0.00)	8 (1.52)	0.72
Dyslipidemia	44 (38.9)	134 (38.6)	20 (40.0)	1 (5.88)	199 (37.8)	0.14
COPD	2 (1.77)	13 (3.75)	0 (0.00)	0 (0.00)	15 (2.85)	0.52
History of CVD	46 (40.7)	116 (33.4)	18 (36.0)	5 (29.4)	185 (35.1)	0.66
PVD	12 (10.6)	27 (7.78)	4 (8.00)	0 (0.00)	43 (8.16)	0.64
Arrhythmia	20 (17.7)	103 (29.7)	14 (28.0)	7 (41.2)	144 (27.3)	0.16
Atrial Fibrillation	8 (7.08)	69 (19.9)	10 (20.0)	5 (29.4)	92 (17.5)	0.04
CAD	20 (17.7)	37 (10.7)	7 (14.0)	0 (0.00)	64 (12.1)	0.21

**Table 2:** Surgical indication table. Nondilated root (NDR), non-syndromically dilated root (NSDR), bicuspid aortic valve (BAV), Marfan’s syndrome (MS), sinus-to-sinus diameter (SSD), Laplace diameter (LD), standard deviation (SD), mean difference (MD).

Patient Group	Coronal SSD (mm)			LD (mm)					Surgery Indication	
	Min	Max	Mean (SD)	Min	Max	Mean (SD)	MD (95% CI) [% Change]	*p*-Value	Coronal SSD Patients (%)	LD Patients (%)
NDR	20.0	39.7	36.3 (3.82)	24.8	51.6	40.4 (5.23)	4.06 (3.17–4.95) [+11.8]	<0.001	0 (0.00)	2 (1.77)
NSDR	34.2	57.0	43.5 (4.06)	31.8	63.8	46.0 (6.27)	2.51 (2.01–3.02) [+5.83]	<0.001	17 (4.90)	87 (25.1)
BAV	31.0	58.0	41.7 (6.2)	32	73.4	45.4 (8.97)	3.75 (1.81–5.68) [+9.25]	<0.001	5 (10.0)	13 (26.0)
MS	35.0	60.0	46.2 (7.5)	38.8	65.6	51.0 (7.58)	4.78 (3.08–6.47) [+10.8]	<0.001	4 (23.5)	9 (52.9)

## Data Availability

The data presented in this study are available on request from the corresponding author as data is not publicly available due to privacy or ethical restrictions.

## Abbreviations

The following abbreviations are used in this manuscript:

TAAD	Thoracic aortic aneurysms and dissections
LD	Laplace diameter
SSD	Sinus-to-sinus diameter
NDR	Nondilated root
NSDR	Non-syndromic dilated root
BAV	Bicuspid aortic valve
MS	Marfan’s syndrome
CT	Computed tomography
ACC	American College of Cardiology
AHA	American Heart Association
